# A pseudohomogeneous nanocarrier based on carbon quantum dots decorated with arginine as an efficient gene delivery vehicle

**DOI:** 10.1038/s41598-021-93153-4

**Published:** 2021-07-02

**Authors:** Aram Rezaei, Ehsan Hashemi

**Affiliations:** 1 Nano Drug Delivery Research Center, Health Technology Institute, Kermanshah University of Medical Sciences, Kermanshah, Iran; 2 Department of Animal Biotechnology, National Institute of Genetic Engineering and Biotechnology, P.O. Box: 14965-16, Tehran, Iran; 3 Diabetes Research Center, Endocrinology and Metabolism Clinical Sciences Institute, Tehran University of Medical Sciences, Tehran, Iran

**Keywords:** Nanobiotechnology, Nanomedicine, Nanoscale materials, Drug delivery

## Abstract

A pseudohomogeneous carrier as an emerging term refers to subnanometric carbon-based vehicle with a high ability to interact with genetic materials to form stable carboplex and successfully transfer them into the cell which will result in inhibiting or expressing of therapeutic genes. Chitosan is a non-toxic polyaminosaccharide used as a precursor in the presence of citric acid to produce carbon quantum dots (CQDs), which decorated with arginine as a surface passivation agent with high amine density in hydrothermal methodology. The Arginine-CQDs are comprehensively characterized by Fourier-transform infrared spectroscopy (FT-IR), Ultraviolet–visible spectroscopy (UV–vis), Atomic force microscopy (AFM), field emission scanning electron microscope (FE-SEM), Energy-dispersive X-ray (EDX) mapping, fluorescence, High-resolution transmission electron microscopy (HR-TEM), zeta potential and X-ray powder diffraction (XRD). In this regard, for the first time, carboplex are formed by electrostatic conjugating of Arginine-CQDs with DNA to protect it from enzymatic degradation. Moreover, the carboplex, like the chitosan precursor, has not shown toxicity against AGS cell line. Interestingly, the Arginine-CQDs have exhibited an excellent ability to overcome cell barriers to deliver into cells compared to chitosan at the same weight ratio. The Arginine-CQDs/pEGFP (W/W) nanocomplex, not only lead to transfection with a relatively higher efficiency than PEI polymer, which is the “golden standard”, but carboplex also demonstrates no significant toxicity. Indeed, the EGFP expression level has reached to 2.4 ± 0.2 via Arginine-CQDs carboplex at W/W 50 weight ratio. To the best of our knowledge, this is the first report includes chitosan-based CQDs functionalized by arginine which is applied to serve as a pseudohomogeneous vehicle for gene transfection.

## Introduction

Gene therapy as a promising new avenue to treat diseases caused by an abnormality in genetic sequence such as cancers, Parkinson’s disease, diabetes and so on, has gained remarkable interest in modern medical science^1–3^. The key point of this treatment is the successful and effective transfer of genetic material through non-toxic nanocarriers to the patient's cell^4,5^. Viral vectors are very popular vehicles in gene therapy due to their high efficiency to deliver small genetic materials. About 70% of clinical trials in gene therapy accomplished based on viral vectors. Nevertheless, this strategy faces different kinds of issues ranging from immunogenicity, toxicity, pathogenicity, carcinogenesis to difficult scaling up. In this regard, non-viral vectors open new opportunities to overcome these difficulties with simple industrial production, biocompatibility and high potential to transport large nucleic acids and high cell specificity^6,7^.

Recently, a collection of diverse artificial non-viral gene carriers such as carbon based materials, dendrimers, liposomes, polymers and inorganic nanomaterial has been investigated for successful transit of genetic materials from several cell barriers and release them into site of action^7^. Among them, polymer-based gene carriers have gained the most interest due to transfection with a high efficiency, but they show moderate toxicity. It has also been observed that some low molecular weight polymers with little toxicity do not form stable complexes with DNA, which will result in low transfection^8^.

Chitosan, as a non-toxic polyaminosaccharide, possesses excellent biomedical application due to its unique physicochemical properties such as mucoadhesiveness, biodegradability and biocompatibility^9–13^. However, the low solubility of chitosan in physiological media has reduced its applications. Therefore, providing innovative chemical methods to increase the solubility and functionality of chitosan and derivatives in aqueous conditions can increase the use of these biocompatible polymers in pharmaceutical and medical. Chemical modification of chitosan can alter the balance between hydrophilic and hydrophobic groups and endowing the cell specificity^14–16^.

CQDs are one of the most encouraging pseudohomogeneous materials with quasi-spherical shapes for cutting-edge technology^17,18^. These zero-dimensional nanomaterials show high fluorescence, biocompatibility, chemical inertness and photostability which can be synthesized based on “Green Chemistry” principles from various carbon sources. By selecting the starting materials and reaction conditions in the bottom-up synthesis of CQDs, the physicochemical properties of the product such as size, edge shape, surface functional groups and defects can be changed for use in the chemical and biological applications^17,18^.

Chitosan is an interesting raw material for the preparation of CQDs due to biocompatibility and high density of NH_2_ and OH groups. Furthermore, the CQDs derived from chitosan have good solubility in various solvents due to sub-nanometric size, high accumulation of different functional groups on the surface of CQDs as well as doping of nitrogen atoms in the carbon lattice which increases the fluorescence nature of CQDs^19,20^. Approaches in order to preparing fluorescent chitosan-based CQDs can be generally classified into two main groups: Hydrothermal and Microwave synthesis methodology^21,22^. Zhao and co-workers synthesized N-doped CQDs from chitosan using hydrothermal carbonization method with a high degree of carbonization. The N content was retained high after treatment at 750 °C, which proved that N atoms doped in the aromatic network of CQDs^23^. Liu’s group fabricated amino-functionalized CQDs derived from chitosan by hydrothermal carbonization. Chitosan-based CQDs without further modification or passivation demonstrate low cytotoxicity with the potential of utilizing as bioimaging agents. In addition, chitosan gel at pH 3 is converted to CQDs under the hydrothermal method. In different strategies, Xiao and co-workers successfully produced fluorescent carbon nitride dots with high solubility in water from chitosan by using of microwave pyrolysis method. This method as an inexpensive and green carbonization approach to produce highly functionalized nanomaterial under mild conditions^22^. Janus’s group prepared CQDs by using of chitosan as a carbon precursor and amino acid as a surface passivation ligand. These functionalized CQDs showed low cytotoxicity and biocompatibility which is a promising material for bioimaging and optoelectronic science^24^. Engineered chitosan-based CQDs with 4-(pyridine-2-yl)-3H-pyrrolo[2,3-c]quinoline (PPQ) to develop nano fluorescence probe to detect a trace amount of water by fast and simple fluorescence method. Photoinduced electron transfer (PET) between pyrrole nitrogen of PPQ and acceptor parts of CDs is the response to increasing fluorescence intensity of probe in the presence of water^25^.

As part of our ongoing research program to explore straightforward methods for the fabrication of functionalized nanomaterials by heterocyclic small molecules^26–32^, herein, for the first time, we wish to report a basic method for the synthesis of chitosan-based CQDs functionalized by arginine groups. Chitosan, citric acid and arginine dissolve in water to produce a homogenous solution, and then, transfer to the autoclave for one-pot hydrothermal synthesis^17,18,33,34^. Arginine is selected as a surface passivation agent with high amine density. In the following, we investigate the ability of positive N-doped CQDs to interact with DNA to form carboplex and the stability of carboplex in physiological and serum media are evaluated. This novel pseudohomogeneous vector exhibit intracellular uptake of EGFP gene to mammalian cells successfully. To the best of the authors’ knowledge, this is the pioneer study on the application of functionalized cationic CQDs derived from chitosan to deliver genetic materials as a novel non-viral gene transfection vector.

## Experimental section

### Materials and characterization

Citric acid, l-arginine, chitosan (Low molecular weight, 50 kDa) and branched-PEI (25 kDa) were purchased from Merck and Sigma. Fetal bovine serum (FBS), dulbecco’s modified Eagle’s medium (DMEM), antibiotics (penicillin and streptomycin), 3-(4,5-dimethylthiazol-2-yl)-2,5-diphenyltetrazolium bromide (MTT) were obtained from Invitrogen. The RNA Isolation Kit was purchased from Roche (Germany). AccuPower RocketScript RT PreMix kit was supplied from Bioneer (Korea, K-2101). The pEGFP-C1 plasmidexpressing enhanced green fluorescent protein (EGFP) was purchased from Clontech Laboratories, Inc. AGS cell line was obtained from National Cell Bank of Iran. The X-ray diffraction (XRD) spectra was conducted on a Siefert XRD 3003 PTS diffractometer with Cu Kα radiation (λ = 1.54 Å). The energy-dispersive X-ray spectroscopy (EDX) analysis and field emission scanning electron microscope (FE-SEM) imaging were measured on a SIGMA VP 500 (Zeiss) microscope. The optical spectra of samples were collected by Shimadzu UV 2100 151PC UV–visible spectrophotometer. Transmission electron microscopy (TEM) images were carried out on Philips EM10C 200 kV microscope. The PerkinElmer PE-1600-FT-IR spectrometer was used to record the fourier-transform infrared spectroscopy (FT-IR) spectra of the samples. The Zeta potentials of the samples were analyzed by using a Zetasizer Nano ZS (Malvern Instruments, Worcestershire, U.K.). The Nikon Eclipse Ti − U inverted microscope was used to observe pEGFP reporter gene expression.

### Fabricating chitosan-based CQDs decorated with arginine (Arginine-CQDs)

The citric acid (1 g) dissolved in 10 mL water and then 1 g chitosan was added slowly with vigorous stirring. After 24 h, arginine (0.3 g) as a passivation agent were dissolved in solution and, stirred for another 2 h. Citric acid acidifies the media so chitosan can be dissolved in the mixture. The obtained solution was hydrothermally treated at 200 °C for 5 h under N_2_ gas in an autoclave. After that, the reddish-brown mixture was centrifuged at 15,000 rpm to remove large particles and then, purified by 100 Da membrane for 48 h.

### Preparation of carrier/pDNA complex and agarose gel retardation assay

The chitosan and Arginine-CQD were separately dissolved in 200 mM acetate buffer (pH = 5.5) and, sonicated for 5 min. Then, solution sterilized by filtration through a 0.22 mm filter. The complex of carrier/pDNA was prepared by adding carrier to 200 ng pDNA at different mass ratio (carrier/pDNA, W/W) from 30:1 to 70:1. The mixture incubated for 30 min at room temperature. 2 μL of loading buffer was added to 8 μL of the mixture and the complexes were electrophoresed onto a 1% agarose gel at 100 V for 60 min in 0.5× TAE (Tris−acetate−EDTA) buffer.

### Measurements of the buffering capacity of chitosan and Arginine-CQD

Acid/base titration was used to determine buffering capacity of chitosan and Arginine-CQD. In this regard, a solution of carriers were diluted to 0.5 mg/mL in deionized (DI) water and titrated with 0.01 M HCl/0.01 M NaOH. The x-axis reported the volume of H^+^ required to changing the pH of solution.

### Cell culture

DMEM with 10% FBS and antibiotics (0.1 mg/mL streptomycin and 100 U/mL penicillin) was supplied as a medium to culture AGS cells at 37 °C in a humidified incubator.

### Cytotoxicity assay

The toxicity of the Arginine-CQD nanocarrier was compared with chitosan and PEI by standard MTT assay. Briefly, AGS cells was seeded onto 96-well plates at 10^4^ cells/well for 24 h in 100 μL of DMEM with 10% FBS. The cells were treated with different mass ratio of carrier/pDNA (W/W) from 30:1 to 70:1 for 24 h. After 5 h, the medium was discard and replaced with fresh medium and cultured for additional overnight. Then, the medium was replaced with MTT solution and cultured for 2 h at 37 °C and 5% CO_2_. The formazan absorbance was quantified at 570 nm. The viability was measured by comparing the percentage of alive cells in the treated wells to the nontreated wells (control).

### In-vitro gene transfection assay

AGS cells were seeded in 96-well plates with density of 10^4^ cells per well and incubated overnight. The cells were treated with different mass ratio of carrier/pDNA (W/W) from 30:1 to 70:1 in FBS-free DMEM. After 4 h. The medium was replaced with complete medium and cultured for further 24 h. The naked pDNA and PEI were used as negative and positive control, respectively. The expression of EGFP was observed by a fluorescence inverted microscope 48 later.

### Total RNA isolation and real-time RT-PCR

The high Pure RNA Isolation Kit was used to extract total RNA in treated and control samples based on manufacturer’s protocol. After synthesis cDNA via AccuPower RocketScript RT PreMix kitand prepared PCR reaction by mixing 8 μL of 2 × SYBR Green PCR Master Mix (Intron, Korea, 25344), 3 μL of RT product, and 0.2 μM of each primer, Real-Time PCR was performed with an ABI System (Applied Biosystems StepOne, USA) under the following thermal conditions: 95 °C for 2 min, and 35 cycles of 95 °C for 10 s and 60 °C for 30 s.

### Statistical analysis

The ANOVA test was used to determine statistical significance. Data have shown as the mean ± standard deviation and *p* values of ≤ 0.05. All stages were repeated at least 3 times.

## Result and discussion

### Preparation and characterization of chitosan-based CQDs decorated by arginine

**Figure 1:**
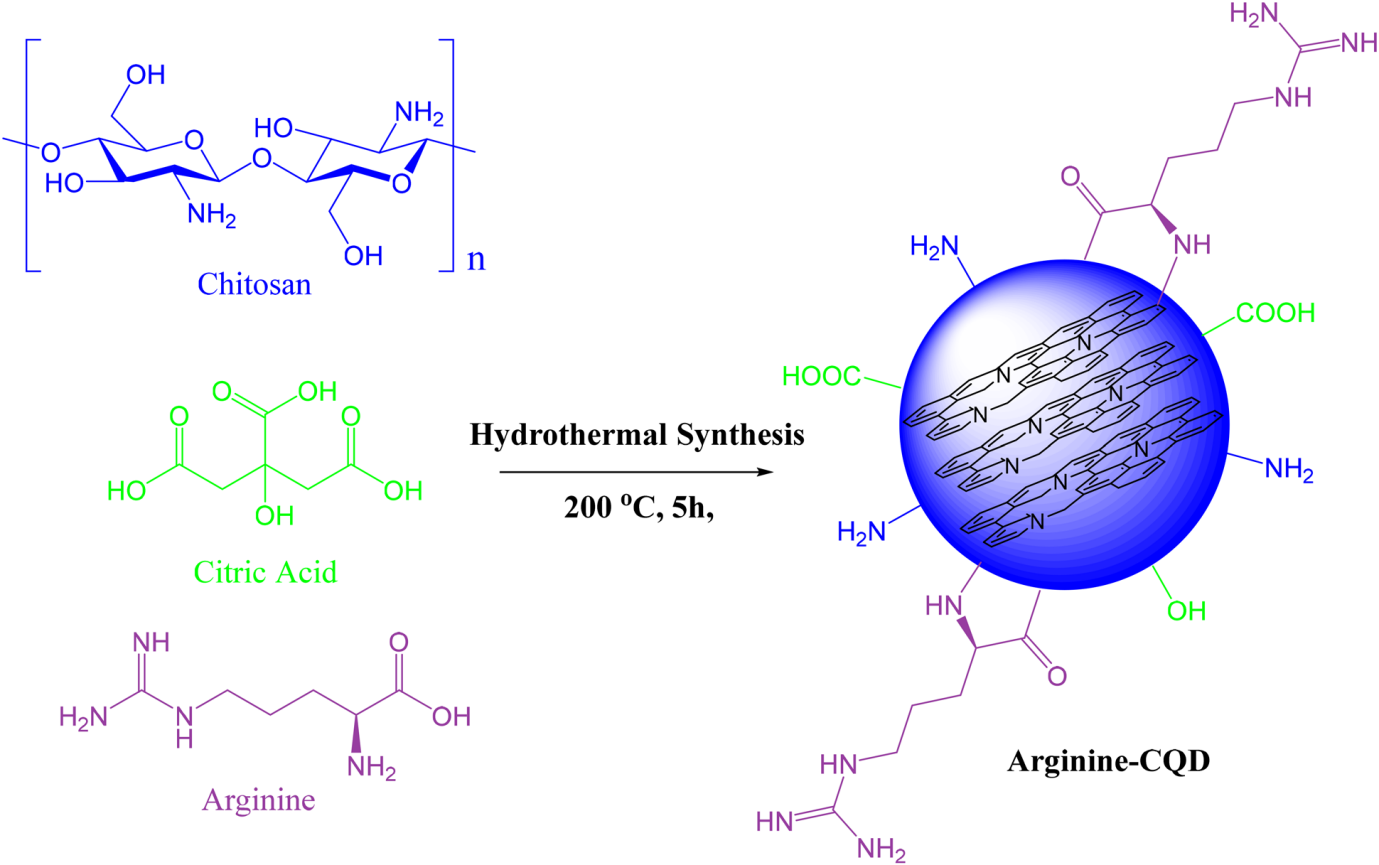
Schematic procedure for the synthesis of the Arginine-CQDs.

The synthetic procedure is illustrated in Fig. 1. Chitosan in the presence of citric acid and arginine underwent hydrothermal treatment at 200 °C for 5 h. starting materials pyrolyzed under the reaction condition to produced cationic CQDs decorated by arginine with high blue fluorescence under UV irradiation (365 nm)^17,18^.

**Figure 2:**
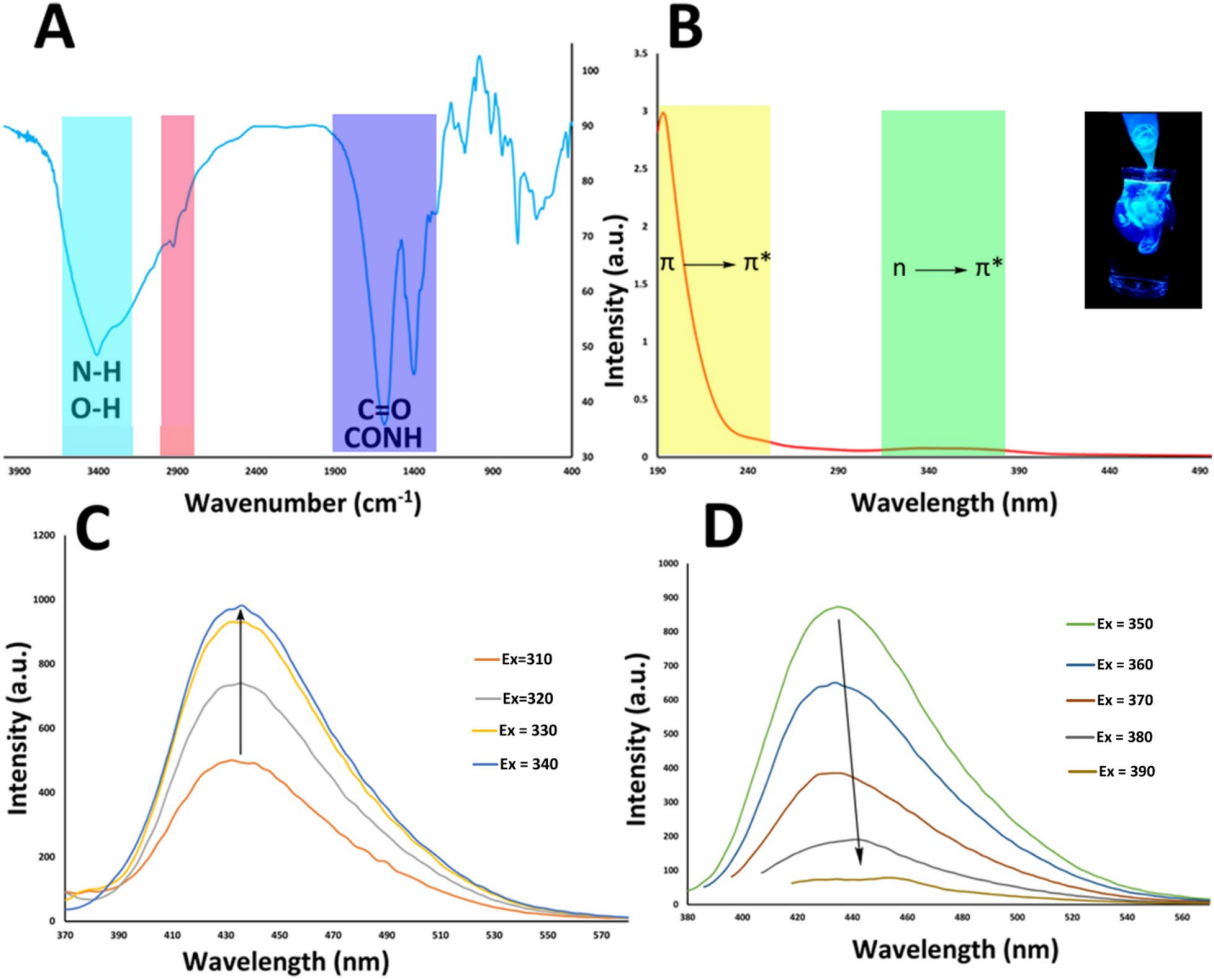
(**A**) FT-IR spectra of the Arginine-CQDs, (**B**) UV–vis absorbance spectra of Arginine-CQDs, the inset shows the emission of Arginine-CQDs dissolved in DI water, (**C**) the emission spectra of Arginine-CQDs with increasing excitation wavelengths from 310 to 340 nm in 10 nm increments, and (**D**) the emission spectra of Arginine-CQDs with increasing excitation wavelengths from 350 to 390 nm in 10 nm increments.

To determine presence of functional groups on the surface of CQDs, FT-IR spectroscopy was used for Arginine-CQDs. The FT-IR spectrum of chitosan exhibit strong bond at 3445 cm^−1^ attributed to O–H and N–H stretches bond^20,21,24^. The C=O stretching and N–H bending of residual N-acetyl groups appear at 1640 and 1590 cm^−1^, respectively. The Arginine-CQDs spectrum was illustrated in Fig. 2A. The bonds located at 3408 and 3260 cm^−1^ are correspond to stretching of N–H, O–H and C–H of aromatic, respectively. Absorptions at 2924–2851 cm^−1^ are assigned to the CH_2_ and CH_3_ stretching bonds. The strong bond at 1590 cm^−1^ is attributed to C=O bond and C=C bond of carbon network. The bonds located at 1400 and 1078 cm^−1^ are associated to N–H and C–O bonds^17,18^. Overall, the Arginine-CQD spectrum shows main expected absorption bonds, which indicates the successful synthesis of this material.

Figure 2B describe the UV–vis spectra of Arginine-CQDs sample. The sample exhibits absorption peaks and shoulder at 194, 247 and 348 nm which can be attributed to aromatic π–π* transition and n–π* transition of aromatic moieties and heteroatom functional groups, respectively^17,18^. As well as, fluorescence spectra for Arginine-CQDs sample is depicted in Fig. 2C,D with increasing wavelengths of excitation in the range of 310 to 390 nm. The chitosan-based CQDs represent the maximum emission at around 435 nm when excited with 340 nm. The stability of fluorescence properties of CQDs vs time, are investigated by examining the change of fluorescence intensity of Arginine-CQDs sample over 3 months^19–21,24^. The results proved that the CQDs derived from chitosan polymer are stable during this period and about 7% of their fluorescence intensity is reduced.

**Figure 3:**
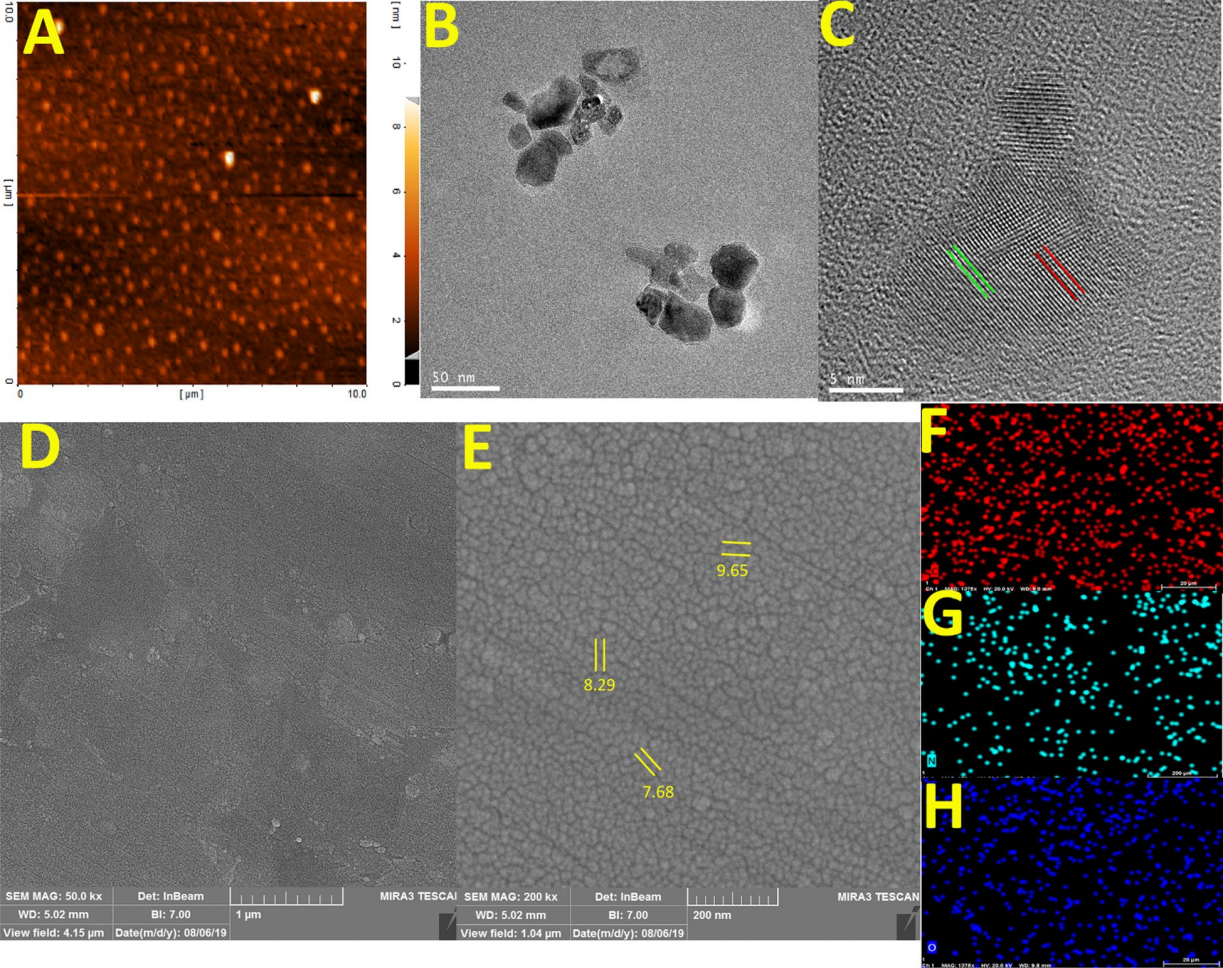
(**A**) The AFM image from Arginine-CQDs, (**B**) TEM image of the of Arginine-CQDs, (**C**) the 5 nm resolution TEM image of Arginine-CQDs, (**D**,**E**) FE-SEM images of Arginine-CQDs, (**F**) the EDX elemental mapping and distribution of C atoms, (**G**) the EDX elemental mapping and distribution of N atoms, and (**H**) the EDX elemental mapping and distribution of O atoms.

Figure 3A illustrate the atomic force microscopy (AFM) image of Arginine-CQDs to investigate dimensional morphology of particles. The AFM technique produces two- and three-dimensional (2D and 3D) images of materials by randomly counting the diameter and height of particles, which present valuable information about surface morphology and topology of CQDs. The uniformity of Arginine-CQDs are good with an average size of seven nm^35–37^. The AFM results show that CQDs particles consist several graphite layers with round disk shape. Further information such as surface morphology, shape and size, monodispersity and crystallinity are also gained by the TEM analysis. The spherical shape, uniformity and average size of particles (6–11 nm) are clearly shown in Fig. 3B^17,18^. The HR-TEM image in Fig. 3C, depicts shape and crystallinity of the Arginine-CQDs, prove that hydrothermally synthesis of CQDs is powerful method to obtain crystalline materials. Finally, these results are in good agreement with AFM analysis.

Figure 3D,E demonstrates the FE-SEM images for as-prepared CQDs. The FE-SEM images of the as-prepared CQDs show spherical shapes with diameters below 10 nm. The sample exhibits monodispersity, which shows the optimal synthesis method and the stability of the particles against aggregation^33,34^. Figures 3F–H show the uniformly distributed of elements achieve by the elemental mapping which show good distribution of elements over the surface of CQDs. These results are in agreement with the outcome achieved by TEM and AFM analysis.

**Figure 4:**
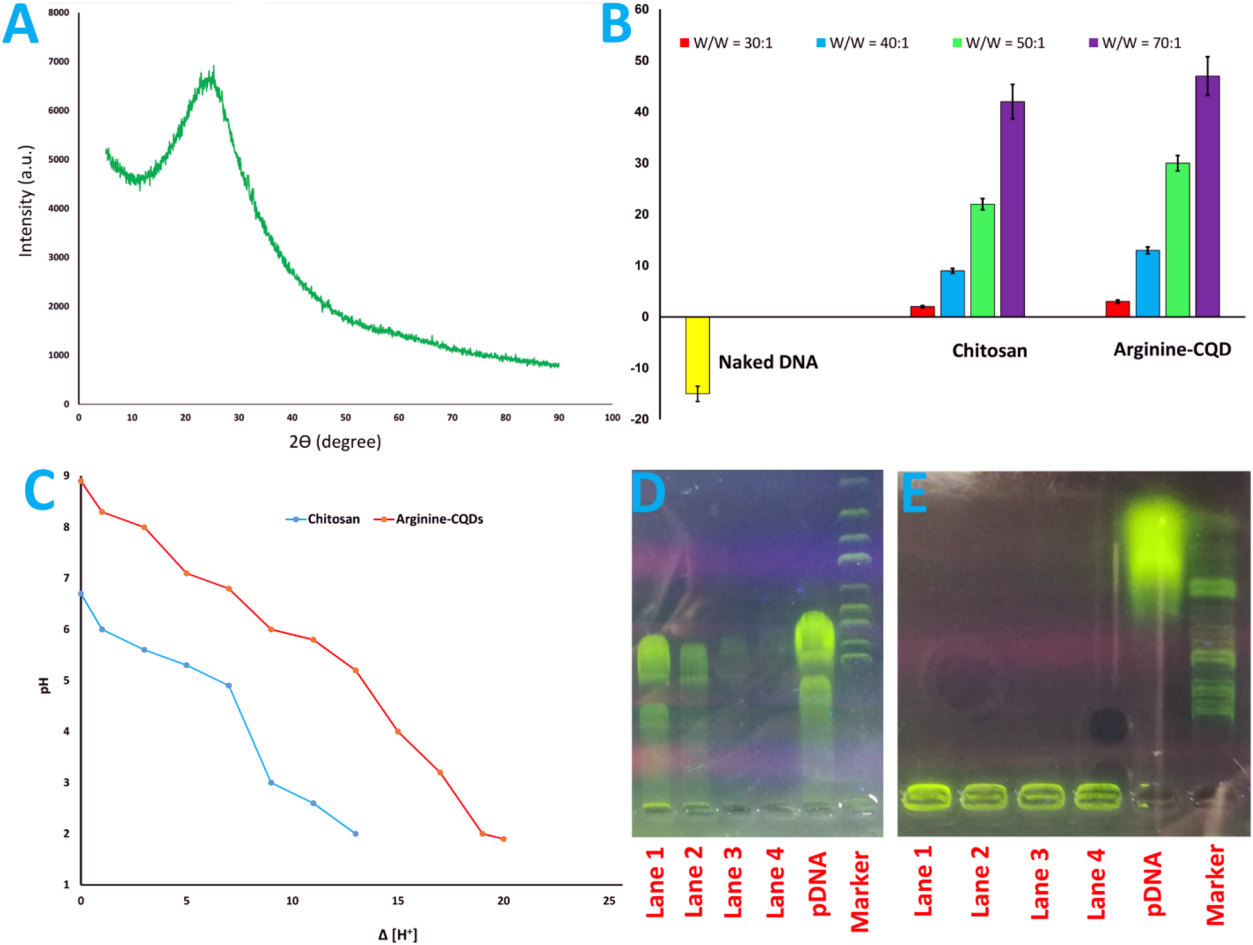
(**A**) The XRD pattern of Arginine-CQDs, (**B**) zeta potential of naked DNA, chitosan, and Arginine-CQDs, (**C**) buffering capacity of chitosan and Arginine-CQDs, (**D**) gel retardation assay of chitosan, (**E**) gel retardation assay of Arginine-CQDs, at various weight ratio. Lane 1, W/W = 30; Lane 2, W/W = 40; Lane 3, W/W = 50; Lane 4, W/W = 70; Lane 5, pDNA; Lane 6, DNA marker.

In addition, after hydrothermal carbonization, the crystalline peak of chitosan at 19.96° was diminished in the XRD pattern and broad peak located at 2θ = 23 attributed to amorphous graphitic-carbon phase of the small size sample was appeared (Fig. 4A)^33^.

### Zeta potential investigation

Zeta potential analysis is used to determine charges on the surface of nanoparticle, which is beneficial for DNA condensation. In this regard, naked DNA, chitosan, and Arginine-CQDs are investigated by zeta potential (Fig. 4B). The chitosan precursor shows positive charge due to presence of large density of amine groups. Consequently, Arginine-CQDs derived from chitosan are also exhibit positive charge. As well as, arginine participates in hydrothermally pyrolysis of citric acid and chitosan to construct graphitic core and functionalized the CQDs surface. Thereby, the proposed nanocarrier show positive charge, which is more desirable to form stable complex with negatively charged DNA^9–13^.

### Buffering capacity

The early escape of nanocarrier/DNA complex from the endosomes is a critical step for successful gene delivery which influenced by buffering capacity of nanovector. Based on proton sponge hypothesis, if the nanocarrier have potential to adsorb protons and buffer lysosomes media, the lysosomal degradation is prevented by swelling of complex and rupture of these vesicle to enter the cell. In this regard, the buffer capacity of chitosan and Arginine-CQDs are measured upon titration with 0.01 N HCl or NaOH^9,38^. Since the pH of the endosomal medium is in the range of 5.5 to 7, the buffering capacity of the materials in this range can help to bursting endosomal vesicle via osmosis and swelling. Figure 4C, demonstrate the buffering capacity of chitosan and Arginine-CQDs. Amine groups of chitosan have p*Ka* value around 6 and in results, chitosan reveal low buffering capacity. Nevertheless, the protonation of chitosan amine groups in acidic media of lysosome is good and cause influx of ions and consequently swelling of endosome and escape from vesicles. Despite chitosan, the Arginine-CQDs exhibit good buffering capacity due to presence kind of amine groups on the CQDs. The primary, secondary and tertiary amine groups are oriented toward the surface of CQDs and are protonated in different pH values. These amine groups are responsible for formation of complex with plasmid, interaction with cell membranes and capturing protons to accelerate early escape from endo/lysosomes^5,7,8,39–41^.

### Formation of CQDs/pDNA complexes

The potency of the Arginine-CQDs as a gene vector to bind with pDNA is investigated by gel retardation assay. The migration of materials in the agarose gel is depend on size, weight and charge. The pDNA movement decreased with addition in nanocarrier/pDNA weight ratios due to increase in size, weight, and decrease negative charge of pDNA/nanocarrier complex. The ability of nanocarriers to complex with DNA is depend on physical interactions between nanocarrier and DNA such as π– π interaction, charge interaction, hydrogen bonding and van der Waals bonds.

This is a critical step in the success of gene therapy. In this regard, the tendency of chitosan polymer and Arginine-CQDs to binding with plasmid, are investigated by gel retardation assay. Chitosan polymer with positive charge form complex with negatively charged DNA^10–13,39^. When the chitosan/plasmid weight ratio was 30:1 or 40:1, the plasmid bond was appeared on the gel, indicating that at these weight ratios, the interaction of the polymer with the plasmid did not result in a stable complex (Figs. 4D and S1). However, by increasing in weight ratio of chitosan/pDNA to 50:1, there is almost no bonds of pDNA (Figs. 4D and S1). Nevertheless, for the Arginine-CQDs case, at any weight ratio, the nanocarrier can form a stable complex with the plasmid (Figs. 4E and S2). Presence of different functional groups on the surface with high density, special architecture and two-dimensional graphene network lead to endow excellent tendency of pseudohomogeneous carrier to bind with DNA. Besides, the DNA fragmentation does not occur due to no extra bonds of DNA are detected in gel electrophoresis proving that the proposed biocompatible nanocarrier meets the standards required to enter a clinical trials^5,6,39,42,43^.

### Cytotoxicity assay of the CQDs

**Figure 5:**
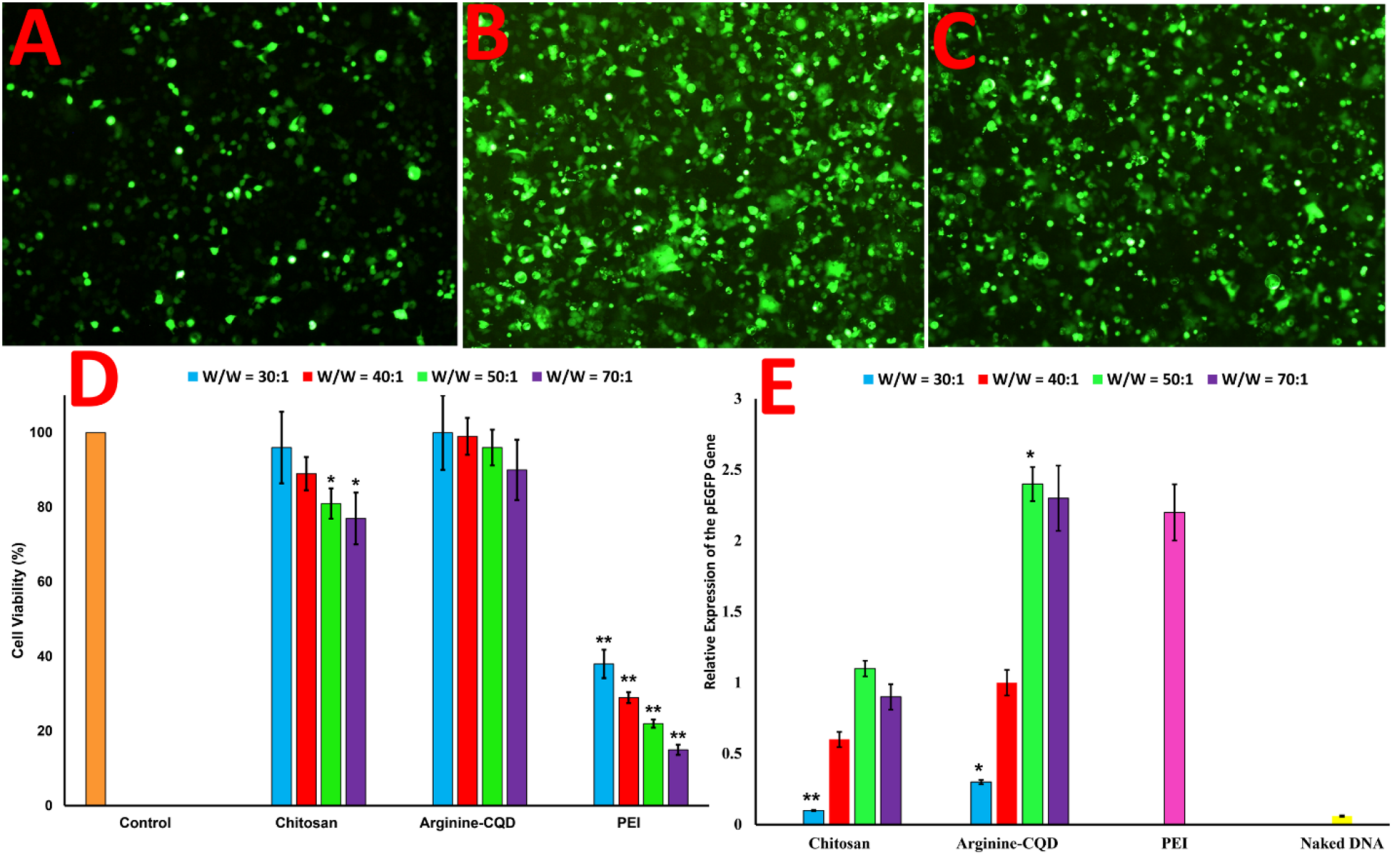
Fluorescence microscopy images of AGS cells transfected with (**A**) chitosan and (**B**) Arginine-CQDs at 50 nanocarrier/pDNA weight ratio (W/W). (**C**) PEI polymer at weight ratio of 1.5, (**D**) cytotoxicity of chitosan, Arginine-CQDs and PEI complex with pEGFP plasmid at W/W ratios of 30, 40, 50 and 70, (**E**) relative gene expression based on real-time PCR for treated cells with chitosan and Arginine-CQDs at W/W ratios of 30, 40, 50 and 70. PEI polymer was used as the positive control (W/W = 1.5) and naked DNA were used as negative control. Data represent the mean ± SD (n = 3, *P < 0.05; **P < 0.01).

Cytotoxicity assay is one of the appropriate methods to determine the toxicity and biocompatibility of carriers^9,12,13^. A non-viral carrier based on CQDs, not only exhibit fluorescence merits and high ability to uptake genetic materials, but also show biocompatibility. In this regard, in-vitro cytotoxicity of chitosan and Arginine-CQDs in the presence of plasmid in various weight ratios from 30:1 to 70:1 (carrier/DNA, W/W) are investigated by MTT assay and is compared with PEI and naked plasmid (Fig. 5D). It is common knowledge, chitosan is a safe polymer and in results, polyplex made with chitosan is approximately nontoxic at moderate mass ratios implemented in gene transfection experiments. In like manner, Arginine-CQDs exhibit negligible toxicity at high concentration, which is proved the biocompatibility of nanocarrier for gene therapy purpose. Even at a W/W of 70, the viability decreased to 90%, while the cell viability value for PEI polymer was only 18%. So, in PEI polymer, toxicity dramatically increases with increment of weight ratio of PEI/DNA. This effect can be due to the high density of amine groups, which induces a large positive charge on polymer. This high positive charge can lead to perturbation of cell membrane by strong electrostatic interaction which will result in decreasing the viability of cells^44^.

### In-vitro transfection of pEGFP into the AGS cells and relative expression of the EGFP

The naked DNA could not cross the cell barriers to uptake into the cells. On the other hand, the systemic toxicity of gene and drug is reduced by using non-viral transferring agent for local administration in the target cells^6,7^. In the present study, the effectiveness of chitosan and Arginine-CQDs are evaluated in transferring pEGFP into AGS cells at various nanovector/pDNA mass ratios (W/W) from 30:1 to 70:1 by fluorescence microscopy and real-time PCR. The PEI as a positive control shows high transfection efficiency to uptake pEGFP into cells at weight ratio of 1.5 due to high affinity to condense with DNA, protecting against enzyme degradation and high buffering capacity. Also, the viability of cell is significantly decreased in the presence of high concentration of PEI polymer (Fig. 5C,E). Chitosan has low buffering capacity with low solubility in physiological pH and its complex with DNA is not very stable, but contrary to expectations, it has a relatively good ability to successfully transfer of genetic material to the cell compared to naked pDNA. By varying W/W ratio of different chitosan weight ranging from 30:1 to 70:1 (chitosan/pDNA), it show high expression of EGFP at a W/W ratio of 50, in which this level reached to 1.7 ± 0.2. Further increment of chitosan/pDNA mass ratio to 70:1 cannot increase the level of EGFP expression (Fig. 5A,E).

The most significant cons of polymer-based nanocarrier are limited to cytotoxicity, solubility, hard scaling up, low efficiency and specificity^44^. Targeting nanocarriers also requires multi-step chemical methods and techniques. In other words, polymer dots as new emerging carbon family have attracted extensive research interest owing to ease of synthesis and modification, fluorescence properties, biocompatibility, huge extent on the starting material, and high solubility in different solvent. Subnanometric size, along with the high density of various functional groups on the surface, make the CQDs as excellent candidate for targeted drug/gene delivery to the cells. The CQDs derived from chitosan show high positive charge and good buffering capacity due to presence amine groups on the surface of CQDs. In addition, the Arginine-CQDs exhibit high dispersity and stability in various pH range.

In this regard, for the first time, we wish to introduce pseudohomogeneous carrier based on arginine functionalized chitosan-based CQDs as powerful carrier to form carboplex with pEGFP and then, transfer successfully genetic materials through cellular barrier to deliver into cells. By addition weight ratio of the Arginine-CQDs, the EGFP expression increases dramatically compared to chitosan by the same weight ratio. The Arginine-CQDs/pEGFP carboplex at mass ratio of 50, not only cause to transfection with a relatively higher efficiency than PEI and reaching EGFP expression level to 2.4 ± 0.2, but also carboplex represent no significant toxicity (Fig. 5B,E).

## Conclusions

The high-efficiency delivery of genetic materials into cells using non-viral targeted vector is one of the most important concerns of researchers in modern medicine. On this account, much research has been directed towards to design and fabrication of carbon nanocarriers to efficient delivery of therapeutic genes and reduce systemic toxicity. In the present study, for the first time, the pseudohomogeneous nanocarrier is introduced with unique architecture that has a high ability to transfer pEGFP to the AGS cells and offer an excellent EGFP expression. The Arginine-CQD carboplex demonstrates excellent gene transfer ability with minimum toxicity in compared to the “gold standard” PEI polyplex. In addition, this cargo illustrates high long-term stability in physiological media. The notable results alongside photostable fluorescence properties of CQDs expand application to real-time tracking and controllable delivery.

## Supplementary Information

Supplementary Information.

## References

[1] Rezaei A, Akhavan O, Hashemi E, Shamsara M (2016). Toward chemical perfection of graphene-based gene carrier via Ugi multicomponent assembly process. Biomacromol.

[2] Xu H, Liao C, Liang S, Ye B-C (2021). A novel peptide-equipped exosomes platform for delivery of antisense oligonucleotides. ACS Appl. Mater. Interfaces.

[3] Mohammadinejad R (2019). Shedding light on gene therapy: Carbon dots for the minimally invasive image-guided delivery of plasmids and noncoding RNAs—A review. J. Adv. Res..

[4] Jones CH, Chen C-K, Ravikrishnan A, Rane S, Pfeifer BA (2013). Overcoming nonviral gene delivery barriers: Perspective and future. Mol. Pharm..

[5] Wiethoff CM, Middaugh CR (2003). Barriers to nonviral gene delivery. J. Pharm. Sci..

[6] De Haan P, Van Diemen FR, Toscano MG (2021). Viral gene delivery vectors: The next generation medicines for immune-related diseases. Hum. Vaccin. Immunother..

[7] Gao X, Kim K-S, Liu D (2007). Nonviral gene delivery: What we know and what is next. AAPS J..

[8] Aied A, Greiser U, Pandit A, Wang W (2013). Polymer gene delivery: Overcoming the obstacles. Drug Discov. Today.

[9] Dehshahri A (2013). Comparison of the effectiveness of polyethylenimine, polyamidoamine and chitosan in transferring plasmid encoding interleukin-12 gene into hepatocytes. Macromol. Res..

[10] Garaiova Z (2012). Cellular uptake of DNA–chitosan nanoparticles: The role of clathrin-and caveolae-mediated pathways. Int. J. Biol. Macromol..

[11] Kritchenkov AS, Andranovitš S, Skorik YA (2017). Chitosan and its derivatives: Vectors in gene therapy. Russ. Chem. Rev..

[12] Chuan D, Jin T, Fan R, Zhou L, Guo G (2019). Chitosan for gene delivery: Methods for improvement and applications. Adv. Coll. Interface. Sci..

[13] Das S, Debnath N, Cui Y, Unrine J, Palli SR (2015). Chitosan, carbon quantum dot, and silica nanoparticle mediated dsRNA delivery for gene silencing in *Aedes aegypti*: A comparative analysis. ACS Appl. Mater. Interfaces..

[14] Abbasian M, Bighlari P, Mahmoodzadeh F, Acar MH, Jaymand M (2019). A de novo formulation of metformin using chitosan-based nanomicelles for potential diabetes therapy. J. Appl. Polym. Sci..

[15] Cao Y, Tan YF, Wong YS, Liew MWJ, Venkatraman S (2019). Recent advances in chitosan-based carriers for gene delivery. Mar. Drugs.

[16] Massoumi B (2020). PEGylated hollow pH-responsive polymeric nanocapsules for controlled drug delivery. Polym. Int..

[17] Hadian-Dehkordi L (2020). Amphiphilic carbon quantum dots as a bridge to a pseudohomogeneous catalyst for selective oxidative cracking of alkenes to aldehydes: A nonmetallic oxidation system. ACS Appl. Mater. Interfaces..

[18] Rezaei A (2021). Pseudohomogeneous metallic catalyst based on tungstate-decorated amphiphilic carbon quantum dots for selective oxidative scission of alkenes to aldehyde. Sci. Rep..

[19] Zattar, A. P. P., Fajardo, G. L., de Mesquita, J. P. & Pereira, F. V. Luminescent carbon dots obtained from chitosan: A comparison between different methods to enhance the quantum yield. *Fuller. Nanotubes Carbon Nanostruct.* 1–10 (2020). 10.1080/1536383X.2020.1854742

[20] Chowdhury D, Gogoi N, Majumdar G (2012). Fluorescent carbon dots obtained from chitosan gel. RSC Adv..

[21] Zhan J (2019). Ethanol-precipitation-assisted highly efficient synthesis of nitrogen-doped carbon quantum dots from chitosan. ACS Omega.

[22] Xiao D, Yuan D, He H, Lu J (2013). Microwave-assisted one-step green synthesis of amino-functionalized fluorescent carbon nitride dots from chitosan. Luminescence.

[23] Zhao L (2010). Sustainable nitrogen-doped carbonaceous materials from biomass derivatives. Carbon.

[24] Janus Ł, Piątkowski M, Radwan-Pragłowska J, Bogdał D, Matysek D (2019). Chitosan-based carbon quantum dots for biomedical applications: Synthesis and characterization. Nanomaterials.

[25] Pawar S, Togiti UK, Bhattacharya A, Nag A (2019). Functionalized chitosan-carbon dots: A fluorescent probe for detecting trace amount of water in organic solvents. ACS Omega.

[26] Massoumi B (2020). A novel bio-inspired conductive, biocompatible, and adhesive terpolymer based on polyaniline, polydopamine, and polylactide as scaffolding biomaterial for tissue engineering application. Int. J. Biol. Macromol..

[27] Adibi-Motlagh B, Lotfi AS, Rezaei A, Hashemi E (2018). Cell attachment evaluation of the immobilized bioactive peptide on a nanographene oxide composite. Mater. Sci. Eng. C.

[28] Ramazani A (2016). Synthesis of 1, 3, 4-oxadiazoles from the reaction of N-isocyaniminotriphenylphosphorane (NICITPP) with cyclohexanone, a primary amine and an aromatic carboxylic acid via intramolecular aza-Wittig reaction of in situ generated iminophosphoranes. Phosphorus Sulfur Silicon Relat. Elem..

[29] Ramazani A (2015). Synthesis of N-acylurea derivatives from carboxylic acids and N,N′-dialkyl carbodiimides in water. J. Chem. Sci..

[30] Ramazani A (2011). Silica gel promotes cascade synthesis of 2-(heteroaryl) acetamide derivatives from isocyanides, dialkylamines, and heteroarylcarbaldehydes. Synth. Commun..

[31] Rezaei A, Ramazani A, Gouranlou F, Woo Joo S (2017). Silica nanoparticles/nanosilica sulfuric acid as a reusable catalyst for fast, highly efficient and green synthesis of 2-(heteroaryl) acetamide derivatives. Lett. Org. Chem..

[32] Rezaei A, Ramazani A, Ahankar H, Ganjeie B, Joo SW (2016). N-Isocyaniminotriphenylphosphorane (Ph3PNNC) as a metal-free catalyst for the synthesis of functionalized isoindoline-1-ones. Phosphorus Sulfur Silicon Relat. Elem..

[33] Mohammadi M, Khazaei A, Rezaei A, Huajun Z, Xuwei S (2019). Ionic-liquid-modified carbon quantum dots as a support for the immobilization of tungstate ions (WO_4_^2–^): Heterogeneous nanocatalysts for the oxidation of alcohols in water. ACS Sustain. Chem. Eng..

[34] Mohammadi M, Rezaei A, Khazaei A, Xuwei S, Huajun Z (2019). Targeted development of sustainable green catalysts for oxidation of alcohols via tungstate-decorated multifunctional amphiphilic carbon quantum dots. ACS Appl. Mater. Interfaces..

[35] Tian X (2020). Carbon quantum dots: In vitro and in vivo studies on biocompatibility and biointeractions for optical imaging. Int. J. Nanomed..

[36] Hu, Q., Gong, X., Liu, L. & Choi, M. M. Characterization and analytical separation of fluorescent carbon nanodots. *J. Nanomater.* **2017** (2017). 10.1155/2017/1804178

[37] Guo D-Y, Shan C-X, Liu K-K, Lou Q, Shen D-Z (2015). Surface plasmon effect of carbon nanodots. Nanoscale.

[38] Tang M, Szoka F (1997). The influence of polymer structure on the interactions of cationic polymers with DNA and morphology of the resulting complexes. Gene Ther..

[39] Lo P-Y (2020). GFP plasmid and chemoreagent conjugated with graphene quantum dots as a novel gene delivery platform for colon cancer inhibition in vitro and in vivo. ACS Appl. Bio Mater..

[40] Pei M, Pai J-Y, Du P, Liu P (2018). Facile synthesis of fluorescent hyper-cross-linked β-cyclodextrin-carbon quantum dot hybrid nanosponges for tumor theranostic application with enhanced antitumor efficacy. Mol. Pharm..

[41] Rabiee N (2021). Polymer-coated NH2-UiO-66 for the codelivery of DOX/pCRISPR. ACS Appl. Mater. Interfaces.

[42] Hasanzadeh A (2021). Photoluminescent carbon quantum dot/poly-l-Lysine core-shell nanoparticles: A novel candidate for gene delivery. J. Drug Deliv. Sci. Technol..

[43] Lin W (2017). Rational design of polymeric nanoparticles with tailorable biomedical functions for cancer therapy. ACS Appl. Mater. Interfaces..

[44] Beyerle A, Irmler M, Beckers J, Kissel T, Stoeger T (2010). Toxicity pathway focused gene expression profiling of PEI-based polymers for pulmonary applications. Mol. Pharm..

