# Supplementary material for: A pseudohomogeneous nanocarrier based on carbon quantum dots decorated with arginine as an efficient gene delivery vehicle
Source: Sci Rep. 2021 Jul 2;11:13790. doi: 10.1038/s41598-021-93153-4 (PMC8253742; doi:10.1038/s41598-021-93153-4)
Supplement: Supplementary file 1 — Supplementary Information. [file 41598_2021_93153_MOESM1_ESM.docx]

*Supporting Information*

**A Pseudohomogeneous Nanocarrier Based on Carbon Quantum Dots Decorated with Arginine as an Efficient Gene Delivery Vehicle**

Aram Rezaei^ɸ*^ and Ehsan Hashemi^‡,†^

ɸ Nano Drug Delivery Research Center, Health Technology Institute, Kermanshah University of Medical Sciences, Kermanshah, Iran

‡ Department of Animal Biotechnology, National Institute of Genetic Engineering and Biotechnology, Tehran, Iran, P.O. Box: 14965-16.

† Diabetes Research Center, Endocrinology and Metabolism Clinical Sciences Institute, Tehran University of Medical Sciences, Tehran, Iran.

Corresponding author: Aram Rezaei, [aram.rezaei@gmail.com](mailto:aram.rezaei@gmail.com), [aram.rezaei@kums.ac.ir](mailto:aram.rezaei@kums.ac.ir)


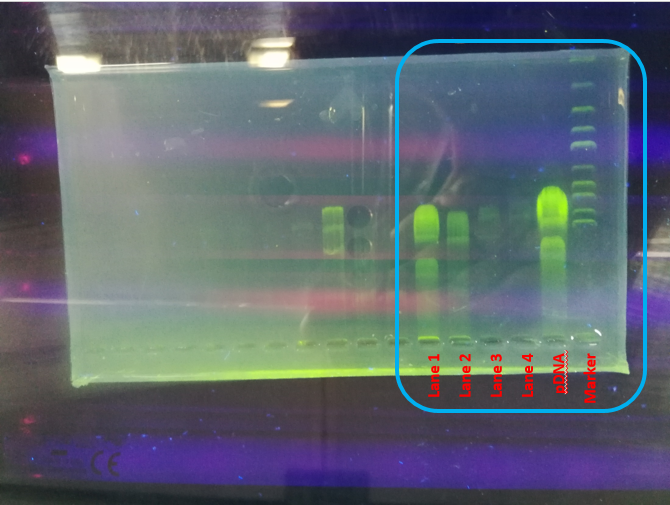


Figure S1: Full length pic for figure 3D in the main text. The blue box is the part that is cut from the original photo. (Gel retardation assay of chitosan at various weight ratio. Lane 1, W/W=30; Lane 2, W/W=40; Lane 3, W/W=50; Lane 4, W/W=70; Lane 5, pDNA; Lane 6, DNA marker).


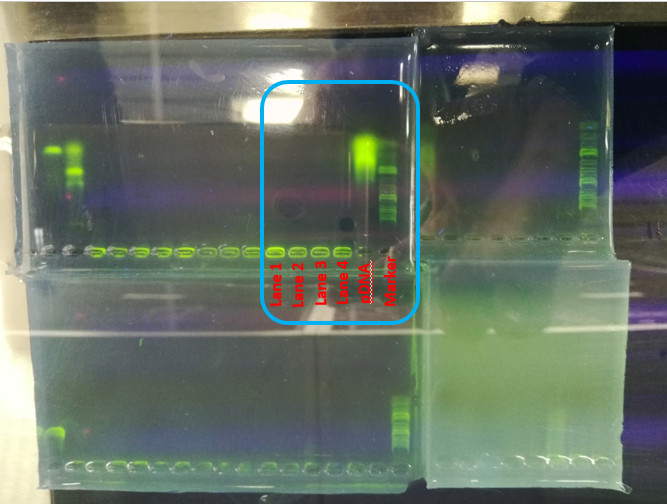


Figure S2: Full length pic for figure 3E in the main text. The blue box is the part that is cut from the original photo. (Gel retardation assay of Arginine-CQDs, at various weight ratio. Lane 1, W/W=30; Lane 2, W/W=40; Lane 3, W/W=50; Lane 4, W/W=70; Lane 5, pDNA; Lane 6, DNA marker).
